# Does perioperative electroacupuncture reduce the incidence of atrial fibrillation after lung cancer surgery? A randomized, controlled, assessor-blinded clinical trial

**DOI:** 10.3389/fmed.2025.1565359

**Published:** 2025-05-12

**Authors:** Zheng-qing Zhou, Yi-jing Chen, Hua Xu

**Affiliations:** Department of Anesthesia, Yueyang Hospital of Integrated Traditional Chinese and Western Medicine, Shanghai University of Traditional Chinese Medicine, Shanghai, China

**Keywords:** postoperative atrial fibrillation, clinical trial, Visual Analog Scale, NT-proBNP, interleukin-6

## Abstract

**Background:**

Atrial fibrillation (AF) is a common complication of lung cancer surgery, with high incidence during the perioperative period. Electroacupuncture is considered a potential complementary therapy for the management of AF. We aimed to evaluate the preventive effects of perioperative electroacupuncture on new-onset AF in patients undergoing lung cancer surgery.

**Methods:**

This was a single-center, randomized, controlled, assessor-blinded clinical trial. We randomly divided 90 patients with cancer who underwent lung surgery into an electroacupuncture group (EA) and a sham electroacupuncture group (SA). Four acupuncture points on the surgical side were selected for the intervention. The EA group was needled with an EA instrument using sparse and dense waves alternating at 2/100 Hz. Electroacupuncture treatments were administered thrice. The incidence of newly developed AF within 72 h after surgery was used as the main indicator, and the Visual Analog Scale, N-terminal pro-B-type natriuretic peptide (NT-proBNP), and blood levels of Interleukin-6 (IL-6) were used as secondary outcomes.

**Results:**

The EA group showed a 14.6% absolute risk reduction in POAF incidence compared to the SA group (*P* = 0.013). NT-proBNP level (MD: +32.57 pg/mL, 95% CI: 5.8–59.3, *P* = 0.018) and interleukin-6 level (MD: +9.29 pg/mL, 95% CI: 1.45–17.1, *P* = 0.021) increased significantly in the SA group compared to the EA group at 72h. No significant differences were observed in VAS scores (12 h: MD −0.27, 95% CI: −0.8 to 0.2, *P* = 0.572; 72h: MD −0.50, 95% CI: −0.4 to 0.3, *P* = 0.238).

**Conclusion:**

This study confirmed that electroacupuncture reduced the incidence of new-onset AF in the perioperative period, providing a possible complementary therapy for the prevention of arrhythmia after lung cancer surgery.

**Clinical trial registration:**

https://www.chictr.org.cn/showproj.html?proj=127920, identifier ChiCTR2100047499.

## 1 Introduction

According to a report by the World Health Organization and the International Union against Cancer, the annual incidence and mortality rates of lung cancer are ranked first globally (1). Lung cancer rates have shown an obvious upward trend in China, with more than 800,000 new cases and 700,000 deaths each year (1). Currently, the preferred treatment option for patients with lung cancer with surgical indications is a comprehensive treatment based on lung cancer surgery (2). However, postoperative complications of lung cancer can be life-threatening, and atrial fibrillation (AF) is a one of common complication after surgery with a 10%–30% rate (3). Postoperative AF (POAF) significantly increases in-hospital stay and costs, has been associated with a variety of adverse cardiovascular events (including stroke, heart failure, and mortality), and significantly increases the risk of recurrent AF in the years after surgery (3, 4). Multicenter studies have further validated the global burden of POAF. For instance, a retrospective, cross-sectional, observational study including 1367 patients reported a 9.4% POAF incidence after lung resection, another study reported that 12.4% of patients experienced POAF, they emphasizing the need for standardized preventive protocols (5, 6). However, it is unclear whether this was due to a causal or simple association (7). Therefore, the prevention of new-onset AF is a key focus in lung cancer surgery.

In the last decade, new data have emerged regarding the pathophysiology of POAF, and new preventive and therapeutic strategies have been proposed and tested in randomized clinical trials (RCTs). Current POAF prevention strategies emphasize risk stratification, electrolyte management (e.g., potassium > 4.0 mmol/L, magnesium > 2.0 mg/dL), and pharmacotherapy. Beta-blockers remain first-line prophylaxis, while amiodarone is reserved for high-risk patients (8, 9). Non-pharmacological approaches, such as posterior pericardiotomy or enhanced recovery protocols, are increasingly adopted in thoracic surgery (9). In order to meet a better reduction of POAF after lung cancer surgery, safer and more effective adjuvant therapies, then entered the research. Acupuncture, a traditional Chinese medicine practice, has also been recognized as having the potential to reduce the incidence of AF. An expert consensus survey demonstrated acupuncture’s efficacy in reducing arrhythmia recurrence across diverse populations (10). Previous studies have suggested that the meaningful mechanism of acupuncture in AF treatment is regulation of the cardiac vagus nerve. A rat model study also confirmed these results and showed that the electroacupuncture (EA) decreased AF inducibility and prevented changes in HRV caused by ACH-CaCl2 injection (11). Researchers believe that acupuncture mediates the balance between sympathetic and vagal activities. Another study reported a significant reduction in left atrial volume index in patients treated with acupuncture after a 12-week treatment period. It demonstrated the role of acupuncture on cardiac remodeling in patients with AF (12). A Meta-analyses showed that there are many acupuncture studies on the prevention of AF after cardiac surgery; however data on the prevention of AF after lung cancer surgery are limited (13). Acupuncture has not been formally tested in adequately powered trials for the treatment of POAF after lung cancer surgery.

Therefore, this study aimed to investigate the clinical effectiveness of electroacupuncture in preventing the incidence of AF after lung cancer surgery through a prospective, randomized, controlled, assessor-blinded clinical trial. In addition to the incidence of POAF, we used NT-proBNP and IL-6, which have recognized roles in predicting atrial remodeling and systemic inflammation (two key pathways in the pathogenesis of POAF), as metrics, with elevated NT-proBNP reflecting myocardial stress, and IL-6 being a key mediator of postoperative inflammation (14, 15). Pain scores were included to assess the potential confounding effect of postoperative discomfort on autonomic tone.

## 2 Methods

### 2.1 Study design and participants

**FIGURE 1:**
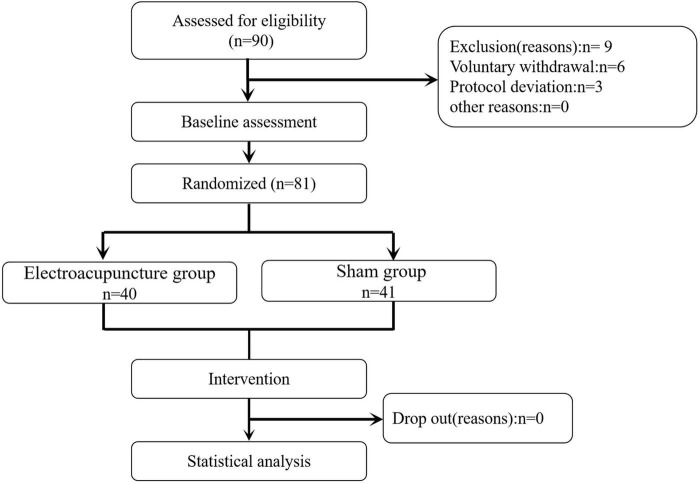
Flow diagram of study design. EA, electroacupuncture.

This study adopted a randomized controlled assessor-blinded clinical trial design. A randomization scheme was prepared by the researcher using the SPSS system, and 90 patients were recruited and divided into two groups: acupuncture and sham acupuncture. The patients were recruited from Shanghai Yueyang Hospital between Jun 2021 and December 2023. The inclusion criteria as followed: (1) Patients with lung tumors who meet the criteria for thoracoscopic lobectomy or pneumonectomy without severe comorbidities, such as cardiac failure (NYHA Class III/IV), chronic kidney disease (eGFR < 30 mL/min/1.73 m^2^), or active cancer metastases. (2) Age not exceeding 85 years, (3) ASA classification I to III, (4) and both sexes are eligible, (5) Completion of admission auxiliary examinations and no history of immune system diseases. (6) Patients who provided written informed consent forms. Patients meeting any of the following criteria were excluded: (1) preoperative arrhythmias, (2) preoperative thyroid dysfunction, (3) use of beta-blockers or digitalis drugs within the past month, (4) history of cardiovascular or thoracic surgery, (5) upper or lower limb nerve injuries, (6) skin infections at the acupoint sites, (7) inability to cooperate with the study plan, including those with language difficulties, infectious diseases, or other medical histories; and (8) participation in other clinical trials within the last 4 weeks. This study was approved by the ethics committee (No. 2021-027) (Figure 1). Trial registration number: ChiCTR2100047499. The roles of the researcher, acupuncturist, clinical anesthesia operator, and statistician were separated in the current study. The participants were informed they might undergo either EA or a noninvasive sham procedure. The outcome assessors and statisticians were blinded to the group allocation throughout the study. The sham acupuncture device mimicked EA activation to minimize participant awareness of their group assignment.

### 2.2 Treatment methods

All patients received EA or sham acupuncture (SA) 30 min before anesthesia induction and until the end of surgery. Two additional treatments were performed at 24 h and 48 h after surgery, each lasting 30 min, using the same acupuncture parameters as described above. The Neiguan (PC-6), Shenmen (HT-7), Hegu (LI-4), and Waiguan (SJ-5) on the surgical side (location strictly follows the Chinese National Standard GB 12346-90 “Location of Acupoints”, and the depth of the needles was 5 mm) were selected as the acupoints of the treatment. All acupuncture related operations were performed by an acupuncturist who has been working at our hospital for more than five years.

#### 2.2.1 EA group

Disposable stainless steel needles (0.25 × 40 mm, Hwato, Suzhou, China) were inserted at PC-6, HT-7, LI-4, and SJ-5 to a depth of 10–25 mm to elicit deqi (a sensation of soreness, numbness, or distension). Electrical stimulation (SDZ-III device, Hwato, Suzhou, China) was applied with alternating sparse-dense waves (2/100 Hz, 5 mA peak current) for 30 min.

#### 2.2.2 SA group

Patients in the SA group were informed that they were receiving a nonperceptible stimulus. Non-penetrating sham needles (Park Device, 0.5 mm blunt tip) were placed at the same acupoints without skin penetration. The device’s LED light mimicked EA activation, but no electrical current was delivered (16).

### 2.3 Sample size calculation

A preliminary trial was conducted with 24 patients (12 per group) undergoing lung cancer surgery between January and April 2020. In this study, the sample size was determined based on a preliminary experiment. The 72h POAF Incidence was used as the assessment index, and it was found that the incidence in the SA group was 41.7% (p1, 5/12), and in the EA group was 16.7% (p2, 2/12). The sample size for comparing two independent proportions was calculated using:


$$n=\frac{{\left(Z_{\alpha /2}\,\sqrt{2\,\bar{p}\,\left(1-\bar{p}\right)}+Z_{\beta }\,\sqrt{p_{1}\,\left(1-p_{1}\right)+p_{2}\,\left(1-p_{2}\right)}\right)}^{2}}{{\left(p_{1}-p_{2}\right)}^{2}}$$

where set the type I error α = 0.05 (one-tailed test, hypothesizing EA reduces POAF risk), type II error β = 0.2, Z_α__/2_ = 1.645, Z_β_ = 0.84. And then considering the 10% dropout rate, it was calculated that 45 subjects were required to be included in each group, for a total of 90 cases in the 2 groups.

### 2.4 Anesthesia and surgery process

All surgeries were performed under general intravenous anesthesia with routine double-lumen endotracheal intubation. The patients were instructed to fast for 6 h and abstain from drinking water for 4 h before surgery. Peripheral and internal jugular venous access was established in the operating room, and lactated Ringer’s solution was infused at a rate of 8–10 ml/kg. Monitoring included electrocardiography, invasive arterial blood pressure, oxygen saturation (SpO_2_), end-tidal carbon dioxide pressure (PETCO_2_), respiratory function, and BIS values. Anesthesia induction involved intravenous administration of propofol (1–2 mg/kg), atracurium (0.15 mg/kg), and sufentanil (5 μg/kg), along with oxygen via a mask and manually assisted ventilation. After muscle relaxation, a double-lumen endotracheal tube of an appropriate size was inserted under laryngoscopic guidance, and its correct position was confirmed by auscultation. Mechanical ventilation was initiated, maintaining the peak airway pressure, PETCO_2_, and SpO_2_ at 15–30 mmHg, 30–35 mmHg, and >93%, respectively. During surgery, continuous infusion of atracurium (0.06 mg/kg/h) and target-controlled infusion of propofol and remifentanil were adjusted to maintain BIS anesthesia. The plasma concentrations of propofol and remifentanil were adjusted to maintain BIS values between 40 and 60, with supplemental sufentanil (0.1 μg/kg) administered as needed. Heart rate and blood pressure were maintained within an acceptable range, and vasoactive drugs were used as needed.

After surgery, all anesthetic drugs were discontinued, and the patients were connected to a patient-controlled intravenous analgesia pump (sufentanil, 2 μg/kg and dezocine 0.3 mg/mL, flow rate, 2 mL/h; bolus dose, 2 mL; lockout time, 15 min). The patients were then transferred to the post-anesthesia care unit (PACU). After returning to spontaneous breathing, neostigmine (2 mg) and atropine (1 mg) were administered to antagonize the muscle relaxants. The endotracheal tube was removed when the extubation criteria were met, and the patients were transferred out of the PACU according to Aldrete’s post-anesthesia recovery score. Within 12 h before surgery and 72 h after surgery, continuous electrocardiographic monitoring was performed using a 24-h dynamic electrocardiogram system (DMS ECG) system.

Basic patient information, including sex, age, preoperative diagnosis, surgical name, surgical site, surgical time, intraoperative anesthesia drug dosages, intraoperative blood loss, intraoperative fluid infusion volume was collected.

### 2.5 Outcomes

#### 2.5.1 Primary outcome

Incidence of newly developed AF within 72 h after surgery

Diagnostic methods and criteria for POAF. All patients undergoing lung resection returned to the thoracic care unit for continuous ECG monitoring, close observing, and recording the time, type, treatment method, and curative effect of AF in detail. If it was difficult to distinguish, bedside electrocardiography was performed. POAF was defined per 2020 ESC criteria (17): irregularly irregular rhythm without discernible P waves, lasting ≥30 s on continuous ECG monitoring or ECG. Stable patients were transferred to an ordinary ward. If patients with heart palpitations; chest tightness; or where physical examination found fast heart rate, heartbeat, or abnormal first heart sound strength, continuous ECG monitoring or urgent check bedside electrocardiogram and AF electrocardiogram was performed, and detailed medical records were kept of the above.

#### 2.5.2 Secondary outcome

N-terminal pro B-type natriuretic peptide (NT-proBNP): BNP levels before surgery and 1 and 3 days after surgery. NT-proBNP level has been proven to be a strong independent predictor of POAF.

Pain Score: Pain scores at 12 and 72 h after surgery were evaluated using the Visual Analog Scale (VAS) pain rating system, with scores ranging from 0 to 10. A score of 0 indicated no pain, and 10 indicated the worst pain. The patients selected a number from the scale to represent their level of pain. The number of times patients pressed the patient-controlled analgesia pump and the need for additional analgesic medications were recorded.

Inflammatory Markers: Blood levels of Interleukin-6 (IL-6) was measured. IL-6 is a pro-inflammatory cytokine, and its elevated levels are proportional to the severity of trauma. It is one of the most sensitive and important biomarkers and mediators of acute stress response in the body.

Blood samples for IL-6 and NT-proBNP measurements were collected preoperatively and at 72 h postoperatively. The samples were centrifuged at 3000 rpm for 10 min, and the plasma was stored at −80°C until analysis. IL-6 levels were measured using a commercially available ELISA kit (R&D Systems, Minneapolis, MN, USA), and NT-proBNP levels were quantified using an electrochemiluminescence immunoassay (Roche Diagnostics, Basel, Switzerland). Both assays were performed in duplicate, and the mean values were used for analysis.

#### 2.5.3 Management of postoperative and acupuncture adverse event

The postoperative adverse reactions included nausea, vomiting, drowsiness, and low oxygen saturation. If the patient fainted or was highly anxious during acupuncture, needling was immediately stopped and the needle should be withdrawn. The patient was placed in the supine position and kept warm. Therefore, researchers should record the adverse events.

### 2.6 Statistical analysis

Data normality was assessed using the Shapiro-Wilk test. For normally distributed variables (e.g., NT-proBNP, IL-6), independent *t*-tests and repeated-measures ANOVA were applied. Non-normally distributed data (e.g., skewed VAS scores) were analyzed using the Mann-Whitney U test. Categorical variables (e.g., POAF incidence) were compared via Fisher’s exact test due to low event rates. All analyses were performed using SPSS 22.0 (IBM Corp., Armonk, NY, USA).

## 3 Results

Table 1 shows the demographic, clinical, and surgical characteristics of the 90 patients. Baseline characteristics of dropouts did not differ significantly between groups. Among the 9 dropouts (EA = 5, SA = 4), the reasons included voluntary withdrawal (*n* = 6), and protocol deviation (n = 3). Table 2 shows a comparison of procedure-related information between the EA group and the SA group, all of which was significantly different (*P* > 0.05 for all variables).

**TABLE 1:** Patient’s characteristics.

Variables	EA group	SA group	*p*-value
	**(*N* = 45)**	**(*N* = 45)**	
Age(year)	65.11 ± 8.65	66.89 ± 8.53	0.329
Male/female	27/18	29/16	0.664
Smoking	12	8	0.310
Hypertension	11	13	0.634
Diabetes mellitus	6	9	0.396
Coronary heart disease	3	5	0.459

**TABLE 2:** The comparison of procedure-related information between the EA group and the SA group.

Variables	EA group	SA group	*p*-value
	**(*N* = 40)**	**(*N* = 41)**	
Preoperative diagnosis (adenocarcinoma/squamous cell carcinoma)	23/17	21/20	0.570
Surgical name (thoracoscopic surgery/open thoracotomy)	36/4	38/3	0.667
Surgical site (left/right)	21/19	16/25	0.224
Operating time (min)	174.30 ± 80.33	193.37 ± 126.48	0.413
**Intraoperative anesthesia drug dosages**			
Propofol (mg)	1085.48 ± 425.42	1218.28 ± 389.84	0.147
Sufentanil (ug)	333.12 ± 45.36	313.68 ± 43.61	0.053
Remifentanil (mg)	2.01 ± 0.36	2.12 ± 0.51	0.283
Atracurium (mg)	20.78 ± 3.32	21.08 ± 5.14	0.696
Bleeding (ml)	150.00 ± 30.71	58.29 ± 39.42	0.295
Intraoperative fluid infusion volume	1302.50 ± 284.19	1287.20 ± 398.71	0.849
Time of discharge	5.93 ± 0.92	5.95 ± 1.0	0.902

**FIGURE 2:**
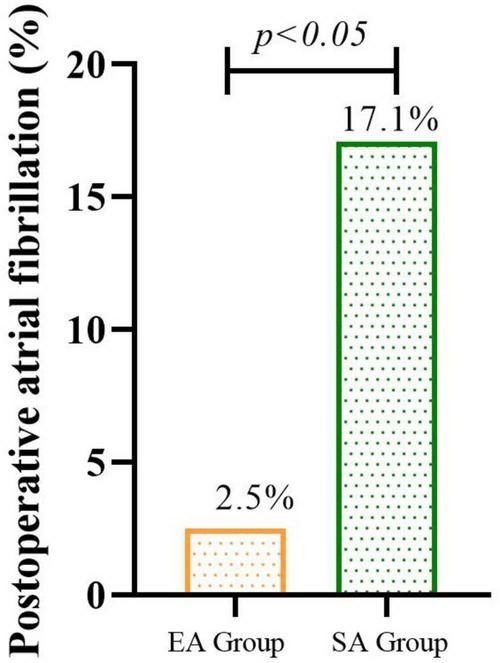
The incidence of new-onset POAF within 72h after surgery in EA groups than in SA group.

Among the remaining participants, the incidence of new-onset POAF within 72 h post-surgery, the EA group (2.5%) showed a 14.6% absolute risk reduction in POAF incidence compared to the SA group (*P* = 0.013) The incidence of new-onset POAF within 72h after surgery was significantly lower in the EA group than in the SA group (Figure 2). Among POAF cases, 6/7 (85.7%) in the SA group occurred within 24h post-surgery, compared to 1/1 (100%) in the EA group. All episodes were paroxysmal; 5 patients received metoprolol, 2 received amiodarone.

**FIGURE 3:**
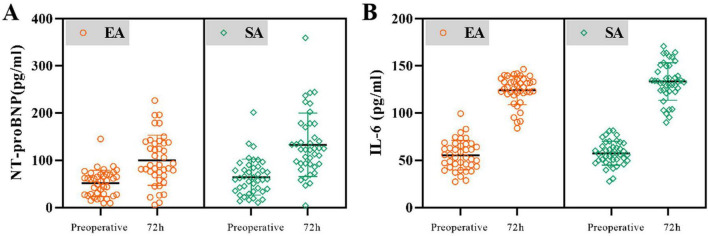
Changes in NT-proBNP **(A)** and IL-6 **(B)** levels during the perioperative course in patients with EA and with SA. Patients before the surgery had significantly lower NT-proBNP levels during the perioperative course as compared to those in 72h after surgery.

Comparison of NT-proBNP and IL-6 levels before and after surgery. There were no significant differences in levels of NT-pro BNP and IL-6 before surgery, and NT-pro BNP 24h after surgery, between the two groups. The NT-pro BNP levels at the end of surgery in the SA group were significantly higher at the 72h after surgery than in the EA group (MD: +32.57 pg/mL, 95% CI: 5.8–59.3, *P* = 0.018). And IL-6 level increased significantly in the SA group compared to the EA group at 72h (MD: +9.29 pg/mL, 95% CI: 1.45–17.1, *P* = 0.021) (Figure 3 and Table 3).

**TABLE 3:** Changes in NT-proBNP and IL-6 levels during the perioperative course in patients with EA and with SA.

Indication	Group	Time		*P*
		**EA group**	**SA group**	
NT-proBNP level	Preoperative	51.06 ± 27.87	64.24 ± 38.27	0.081
	24 after the surgery	173.80 ± 76.23	196.53 ± 90.94	0.227
	72 h after the surgery	100.01 ± 53.09	132.58 ± 66.95	0.018
IL-6	Preoperative	55.44 ± 15.66	57.48 ± 12.54	0.519
	72 h after the surgery	123.94 ± 15.17	133.22 ± 19.88	0.021

**FIGURE 4:**
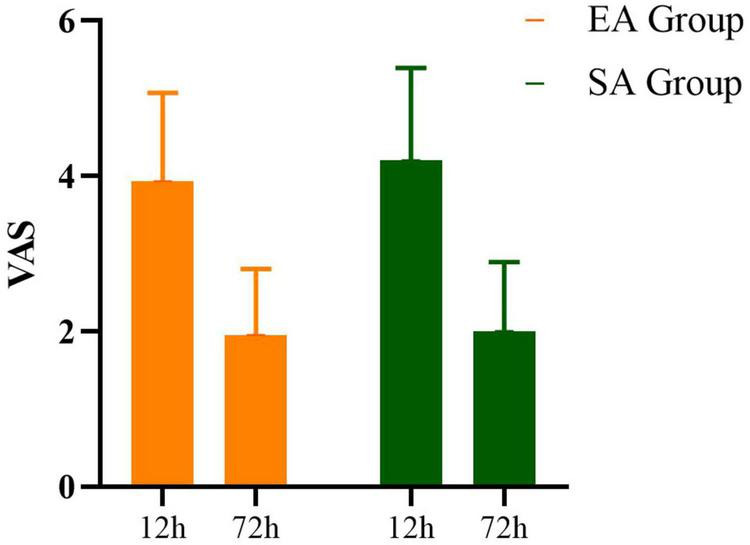
Changes in VAS during the perioperative course in patients with EA and with SA.

Postoperative pain status in the two groups of patients. There were no significant differences in the VAS scores between the two groups at 12 (MD −0.27, 95% CI: −0.8 to 0.2, *P* = 0.572) and 72 h (MD −0.50, 95% CI: −0.4 to 0.3, *P* = 0.238) after surgery (*P* > 0.05) (Figure 4 and Table 4).

**TABLE 4:** Changes in scores of VAS during the perioperative course in patients with EA and with SA.

VAS	Time		*p*
**Group**	**EA group**	**SA group**	
12 h after the surgery	3.03 ± 1.19	2.80 ± 1.14	0.572
72 h after the surgery	1.33 ± 0.86	1.56 ± 0.32	0.238

The mean number of analgesic pump presses within 72h post-surgery was 5.3 ± 2.2 (EA) vs. 5.7 ± 1.8 (SA) (*P* = 0.422). Additional analgesics were required in 3/40 (7.5%) EA patients and 5/41 (12.2%) SA patients (*P* = 0.468). No patients experienced postoperative adverse reactions such as drowsiness, and low oxygen saturation, but 7 patients (EA = 4, SA = 3) experienced nausea and vomiting, 17 patients (EA = 7, SA = 10) experienced fatigue, and 15 patients (EA = 7,SA = 8) experienced cough. And, there were no complaints of bleeding, hematoma, or infection due to acupuncture treatment. No pain or vasovagal reactions were reported after needle insertion.

## 4 Discussion

To our knowledge, this is the first prospective, assessor-blinded, randomized trial to investigate the effects of EA treatment on the incidence of POAF after lung cancer surgery. Our study demonstrated that perioperative EA significantly reduces the incidence of new-onset POAF in patients undergoing lung cancer surgery, with a 14.6% absolute risk reduction compared to SA. The lower NT-proBNP levels in the EA group at 72 h postoperatively (*P* = 0.018) suggest attenuated myocardial stress, potentially linked to EA’s sympatholytic effects (18). These results are consistent with prior trials in cardiac surgery populations (19, 20). However, unlike previous reports emphasizing EA’s analgesic benefits, we observed no significant differences in VAS scores, possibly due to standardized postoperative analgesia masking EA’s adjunctive effects. This underscores the need to evaluate EA’s arrhythmia-specific mechanisms independently of pain modulation.

In lung cancer surgery, POAF is particularly prevalent due to the interplay of surgical trauma, pulmonary resection-induced inflammation, and autonomic nervous system disruption. Thoracic procedures, such as lobectomy or pneumonectomy, directly irritate the pericardium and pulmonary veins, creating a proarrhythmic substrate. Additionally, postoperative hypoxia, electrolyte imbalances (e.g., hypokalemia), and systemic inflammation from surgical stress further exacerbate atrial electrical instability (21–23). Lung cancer patients often exhibit elevated baseline inflammatory markers (e.g., IL-6, C-reactive protein) due to tumor-associated inflammation, which may amplify postoperative inflammatory responses and increase POAF risk (15). These factors highlight the unique vulnerability of lung cancer surgery patients to POAF and underscore the importance of targeted preventive strategies like electroacupuncture, which modulates both autonomic activity and inflammation.

The generalizability of our findings warranted cautious interpretation. As a single-center trial with strict inclusion criteria (ASA I–III), our cohort excluded high-risk subgroups (e.g., severe comorbidities), limiting extrapolation to broader populations. Additionally, EA’s efficacy may depend on practitioner expertise, as interventions were delivered by a single experienced acupuncturist. Cultural factors also play a role: in China, patient acceptance of acupuncture likely enhanced blinding efficacy for SA, whereas skepticism in other regions might compromise trial validity. Future multicenter studies should standardize EA protocols across diverse settings and include long-term follow-up to assess POAF recurrence. Mechanistic investigations—such as heart rate variability (HRV) monitoring and expanded biomarker panels (e.g., TNF-α, CRP)—are critical to elucidate whether EA’s benefits stem from autonomic rebalancing, anti-inflammatory effects, or a combination of both. Subgroup analyses stratified by surgical approach (lobectomy vs. pneumonectomy) or cancer stage could further refine its clinical utility.

Despite these limitations, EA presents a compelling safety profile, with no serious adverse events reported. Minor side effects (e.g., nausea, fatigue) were comparable between groups, supporting its feasibility in perioperative care. However, scalability challenges persist: EA requires specialized equipment, trained personnel, and additional time, which may strain resources in high-volume centers. Cost-effectiveness analyses are needed to justify its integration into enhanced recovery protocols. Furthermore, while SA served as an active control, comparisons to standard care (no acupuncture) would clarify whether observed benefits reflect specific EA effects or nonspecific placebo responses. Balancing these considerations, our study provides robust preliminary evidence for EA as a complementary strategy to mitigate POAF risk in lung surgery, particularly in settings where pharmacological prophylaxis is contraindicated or insufficient. Collaborative efforts between anesthesiologists, surgeons, and traditional medicine practitioners will be essential to optimize EA’s role within multimodal perioperative frameworks.

The following limitations of this study must be considered. This study utilized a sham-controlled design rather than a static group (no intervention) to preserve blinding and ethical standards. While this approach isolates the specific effects of EA, it limits direct comparison to routine care alone. Future trials could incorporate a three-arm design (EA, sham, and static) to further elucidate the intervention’s efficacy relative to natural POAF incidence. The present study lack of long-term follow-up for AF recurrence. Therefore, the treatment effects may be different in these patients. Continuous electrocardiographic monitoring was used during the perioperative period to detect every episode of POAF. However, indicators such as NT-proBNP and IL-6 levels were not collected immediately after the occurrence of POAF, and the reported treatment effect might be lower. This may explain the lack of significant differences in these indicators.

## 5 Conclusion

In this preliminary trial, perioperative electroacupuncture was associated with a reduced incidence of POAF after lung cancer surgery. While promising, these findings require validation in larger, multicenter studies. Standardized protocols for acupuncture in POAF prevention should be explored in future research. This study provides new strategies for preventing new-onset AF after lung cancer surgery.

## Data Availability

The raw data supporting the conclusions of this article will be made available by the authors, without undue reservation.

## References

[1] Sung H Ferlay J Siegel R Laversanne M Soerjomataram I Jemal A Global cancer statistics 2020: GLOBOCAN estimates of incidence and mortality worldwide for 36 cancers in 185 countries. *CA Cancer J Clin.* (2021) 71:209–49. 10.3322/caac.21660 33538338

[2] Montagne F Guisier F Venissac N Baste J. The role of surgery in lung cancer treatment: Present indications and future perspectives-state of the art. *Cancers (Basel).* (2021) 13:3711. 10.3390/cancers13153711 34359612 PMC8345199

[3] Gaudino M Di Franco A Rong L Piccini J Mack M. Postoperative atrial fibrillation: From mechanisms to treatment. *Eur Heart J.* (2023) 44:1020–39. 10.1093/eurheartj/ehad019 36721960 PMC10226752

[4] LaPar D Speir A Crosby I Fonner E Brown M Rich J Postoperative atrial fibrillation significantly increases mortality, hospital readmission, and hospital costs. *Ann Thorac Surg.* (2014) 98:527–33. 10.1016/j.athoracsur.2014.03.039 25087786

[5] Crispi V Isaac E Abah U Shackcloth M Lopez E Eadington T Surgical factors associated with new-onset postoperative atrial fibrillation after lung resection: The EPAFT multicentre study. *Postgrad Med J.* (2022) 98:177–82. 10.1136/postgradmedj-2020-138904 33310899

[6] Onaitis M D’Amico T Zhao Y O’Brien S Harpole D. Risk factors for atrial fibrillation after lung cancer surgery: Analysis of the society of thoracic surgeons general thoracic surgery database. *Ann Thorac Surg.* (2010) 90:368–74. 10.1016/j.athoracsur.2010.03.100 20667313

[7] van Boven W de Groot J Kluin JA. short cut to prevent postoperative atrial fibrillation. *Lancet.* (2021) 398:2052–3. 10.1016/S0140-6736(21)02435-1 34788639

[8] Wang X Zhang D Ren Y Han J Li G Guo X. Pharmacological interventions for preventing atrial fibrillation after lung surgery: Systematic review and meta-analysis. *Eur J Clin Pharmacol.* (2022) 78:1777–90. 10.1007/s00228-022-03383-2 36136141

[9] Van Gelder I Rienstra M Bunting K Casado-Arroyo R Caso V Crijns H 2024 ESC guidelines for the management of atrial fibrillation developed in collaboration with the European association for cardio-thoracic surgery (EACTS). *Eur Heart J.* (2024) 45:3314–414. 10.1093/eurheartj/ehae176 39210723

[10] Li J Wang L Zhang N Su X Lin Y Yang J Acupuncture as an adjunctive therapy for arrhythmia: A Delphi expert consensus survey. *Cardiovasc Diagn Ther.* (2021) 11:1067–79. 10.21037/cdt-21-201 34815957 PMC8569262

[11] Su Y Huang J Sun S He T Wang T Fan M Restoring the autonomic balance in an atrial fibrillation rat model by electroacupuncture at the neiguan point. *Neuromodulation.* (2024) 27:1196–207. 10.1016/j.neurom.2022.11.005 36522251

[12] Lee J Lee S Leem J Kim J Park J Park J Effects of acupuncture on cardiac remodeling in patients with persistent atrial fibrillation: Results of a randomized, Placebo-Controlled, patient- and Assessor-Blinded pilot trial and its implications for future research. *Medicina (Kaunas).* (2021) 58:41. 10.3390/medicina58010041 35056349 PMC8778603

[13] Li Y Song J Wu B Wang X Han L Han Z. Acupuncture versus pharmacological conversation in treatment of atrial fibrillation in a randomized controlled trial: A systemic review and meta-analysis. *Eur J Med Res.* (2022) 27:110. 10.1186/s40001-022-00738-4 35786416 PMC9252049

[14] Wang W Zhou T Li J Yuan C Li C Chen S Association between NT-proBNP levels and risk of atrial fibrillation: A systematic review and meta-analysis of cohort studies. *Heart.* (2025) 111:109–16. 10.1136/heartjnl-2024-324685 39643423

[15] Jia X Cheng X Wu N Xiang Y Wu L Xu B Prognostic value of interleukin-6 in atrial fibrillation: A cohort study and meta-analysis. *Anatol J Cardiol.* (2021) 25:872–9. 10.5152/AnatolJCardiol.2021.69299 34866581 PMC8654009

[16] Fan W Ma W Zhao C Tong Q Shen W. [Influence of acupuncture-drug compound anesthesia with different frequency electroacupuncture on immune function in patients undergoing pneumonectomy]. *Zhongguo Zhen Jiu.* (2012) 32: 715–9. 10.13703/j.0255-2930.2012.08.020 23072093

[17] Hindricks G Potpara T Dagres N Arbelo E Bax J Blomström-Lundqvist C 2020 ESC guidelines for the diagnosis and management of atrial fibrillation developed in collaboration with the European Association for Cardio-Thoracic Surgery (EACTS): The task force for the diagnosis and management of atrial fibrillation of the European society of cardiology (ESC) Developed with the special contribution of the European heart rhythm association (EHRA) of the ESC. *Eur Heart J.* (2021) 42:373–498. 10.1093/eurheartj/ehaa612 32860505

[18] Lombardi F Belletti S Battezzati P Lomuscio A. Acupuncture for paroxysmal and persistent atrial fibrillation: An effective non-pharmacological tool? *World J Cardiol.* (2012) 4:60–5. 10.4330/wjc.v4.i3.60 22451853 PMC3312232

[19] Feingold K Moskowitz J Elenbaas C Andrei A Victorson D Kruse J Acupuncture after valve surgery is feasible and shows promise in reducing postoperative atrial fibrillation: The ACU-Heart pilot trial. *JTCVS Open.* (2023) 16:321–32. 10.1016/j.xjon.2023.05.010 38204624 PMC10774881

[20] Liu Y Pang X Wang Y Liu X Jiang H. Evaluation of the efficacy and safety of acupuncture assisted treatment for atrial fibrillation: A systematic review and meta-analysis based on randomized controlled trials. *Medicine (Baltimore).* (2024) 103:e40474. 10.1097/MD.0000000000040474 39612438 PMC11608700

[21] Alam N Park B Wilton A Seshan V Bains M Downey R Incidence and risk factors for lung injury after lung cancer resection. *Ann Thorac Surg.* (2007) 84:1085–91. 10.1016/j.athoracsur.2007.05.053 17888952

[22] Furák J Németh T Lantos J Fabó C Géczi T Zombori-Tóth N Perioperative systemic inflammation in lung cancer surgery. *Front Surg.* (2022) 9:883322. 10.3389/fsurg.2022.883322 35669251 PMC9163434

[23] Luo X Ying Y Yin L Chang P. Analysis of risk factors for hypoxemia in PACU for patients undergoing thoracoscopic lung cancer resection based on logistic regression model. *BMC Anesthesiol.* (2025) 25:174. 10.1186/s12871-025-03043-9 40217167 PMC11987176

